# Cell Regulation of Proliferation and Differentiation ex vivo for Cells Containing Ph Chromosome in Chronic Myeloid Leukemia

**Published:** 2009-10

**Authors:** N.I. Grineva, T.V. Akhlynina, L.P. Gerasimova, T.E. Manakova, N.G. Sarycheva, D.A. Schmarov, A.M. Tumofeev, N.M. Nydenova, L.Yu Kolosova, T.I. Kolosheynova, L.G. Kovaleva, S.V. Kuznetsov, A.V. Vorontsova, A.G. Turkina

**Affiliations:** 1 GU National Research Center for Hematology, Russian Academy of Medical Sciences

## Abstract

Cell regulation of Ph^+^cell proliferation and differentiation has been studied ex vivo in various chronic myeloid leukemia (CML) patients. The regulation is provided by alternation of effective stages of proliferation and maturation with inhibition of Ph^+^ cell proliferation by accumulating neutrophils under apoptosis blockage. The alternation of stages consists of switching stage 1 (effective proliferation) to stage 2 (effective maturation) and proceeds according to the 1/2 -1/2/1 or 2/1-2/1/2/1 schemes. The kinetic plots of alternations pass through control points of crossing plots, where the parameters of proliferation and maturation are equal. The indices of P/D efficiency (ratio of proliferation and maturation rates) are 1.06±0.23 and don't depend on time, alternation order, or sources of Ph^+^ cells - CML patients. During stages alternation, conversely, the parameters of Ph+ cell proliferation and maturation vary. The proliferation stages are characterized by increased proliferating cells content, a decreased number of neutrophils, and apoptosis induction. At the maturation stages, conversely, apoptosis is inhibited, the number of mature neutrophils increases, while immature Ph^+^ cells decrease. High content neutrophils inhibit the proliferation of Ph^+^ cells and impair their own maturation by inversion of maturation order, probably through a feedback mechanism. The regulation differences ex vivo reveal three types of Ph^+^ cells from various individual CML patients, distinguished by the number and duration of alternating stages of proliferation and maturation. Ph^+^ cells types 1 and 2 have one prolonged stage of effective proliferation or effective maturation with efficiency indices P/D^1^ = 1-20 or P/D^2^ ⇐ 1. At the same time period, the proliferation and differentiation of the Ph^+^ cells type 3 proceeds with repeated alternations of stages with P/D^1^ = 1-4 or P/D^2^ ⇐ 1. Type 1 Ph^+^ cells (~20%) were isolated from patients in advanced stages of CML, while Ph^+^ cells types 2 and 3 (30 and 50% correspondingly) were isolated from CML chronic phase patients sensitive to chemotherapy.

## INTRODUCTION

Leukemias accounts for 1% of all deaths and 4-10% of deaths from cancer. The prevalence of leukemias and lymphomas varies from 3 to 9:100 000, depending on the geographical region. Unfavorable radiation and ecological environment can increase it by 1.5 logs. In the U.S., leukemias are the major reason of death in children before 15.

The majority of leukemias result from genetic disturbances: chromosomal aberrations, translocations, inversions, deletions, and various mutations [1-3, 6].

Philadelphia-positive (Ph^+^) cells, expressing active tyrosine kinase p210 or p185 (oncoproteins, products of bcr/abl gene), are involved in chronic myeloid leukemia (CML) pathogenesis. It results in reciprocal chromosomal translocation t(9;22)(q34;q11) in the polipotent hematopoietic stem cell. Proliferation and differentiation of this cell leads to replacement of normal hematopoietic cells by their monoclonal neoplastic Ph^+^ counterparts, thus promoting the development and progress of CML [1-8, 10,12].

The CML clinical course varies among different patients. The cellular and molecular mechanisms of these differences remain unclear. Current knowledge of CML course and progression in vivo is based upon analyses of averaged values of various parameters obtained at different moments and CML phases. CML undergoes a chronic phase (CP), accelerated phase (AP), and an acute rapidly progressing blast phase (BP) with an inevitable fatal outcome. Current CML therapy is based upon highly specific targeted drugs, tyrosine kinase inhibitors (TKI), specifically blocking p210 - imatinib and its analogues. Imatinib allows to extend life by 6 years in 88% of patients., of which 66% continue treatment. In 14% of those patients, CML progresses, while 5% of them interrupt treatment because of toxicity. The toxicity is associated with additional bcr/abl gene mutations, leading to therapeutic resistance. Despite the development of a new generation of TKIs, the problem remains unsolved, because none of them kills the resting leukemia stem cells. Fewer than 5% of CML chronic phase patients are cured, while the majority eventually relapse [6]. There is a need for another strategy in dealing with leukemia stem cells.

Despite the extensive research of Ph^+^ cells both in cultures and in vivo [4, 5, 7-12, 15-20,23,27], the processes taking place in the cells of newly diagnosed CML patients and in those in progression remain poorly studied. There is no unified conception of the biological and molecular processes underway in CML both in vitro and in vivo and their interaction. Little is known about the patterns of proliferation and differentiation (PAmp;D) of Ph^+^ cells even in vitro.

Researchers have often noted that the cellular processes in cells isolated from CML patients differ from those in cell lines. The number of proliferating hematopoietic progenitors in CML is decreased, while the number of nondividing mature neutrophils is higher than in normal cells [13,14,20-23,26]. Ph^+^ stem cells proliferate less actively than healthy donor stem cells, while more differentiated Ph^+^ cells are accumulating more actively [20, 23, 24, 27]. This leads to suggest that the cause of CML is the imbalance between stem cells self-renewal and myeloid progenitors maturation, rather than Ph^+^ cells proliferation under the action of p210 ^bcr/ abl^ , as follows from many other studies [4-7,10-12, 15, 16, 19, 24-29, 31].

It should be mentioned that the question of apoptosis block in Ph^+^ cells is also not obvious. Evidently, our knowledge of cellular and molecular regulation of Ph^+^ cells in CML is still incomplete, though the oncogenic potential of bcr/abl oncogene, including enhanced cell viability, proliferation activation, and resistance to apoptosis, is studied in details in various studies [7, 4,17,18, 25-27, 29, 30, 35 ,43-45].

The influence of different p210 mutations on proliferation, apoptosis, and the malignant properties of Ph^+^ cells transfected with different bcr/abl mutated variants was studied in [27]. The activation of proliferation and apoptosis inhibition by p210 was shown to be independent processes closely linked to different bcr/abl mutations (including those responsible for intracellular signaling via various STAT proteins). It was hypothesized that those mutations can influence the severity of proliferation activation and apoptosis inhibition and shift their relative impact. In Ph^+^ cells of CML patients, these properties are not studied due to lack of suitable models and experimental approaches, though the bcr/abl mutations are actively studied and used in practice for CML diagnosis and treatment optimization [19, 29, 30-32]. Based on these considerations, we have suggested that the kinetics of Ph^+^ cells PAmp;D can reveal the differences in Ph^+^ cells PAmp;D regulation of individual patients ex vivo.

The goal of our study was to clarify the mechanism of Ph^+^ cells PAmp;D in individual CML patients ex vivo in suspension cultures by means of the previously developed kinetic method of study [33, 39]. The pattern of CML cells proliferation and differentiation in cultures is similar to that of enhanced myelopoiesis in CML in vivo, allowing to estimate the efficiency of PAmp;D, influence of growth factors, and the expression of bcr/abl and differentiation antigens in Ph^+^ cells.

## MATERIALS | METHODS

Materials: heparin (Flow, UK); Limphoprep, alpha-MEM media (MP Biomedical, USA); DEPC, Hepes, Tris, PBS, fetal bovine serum (FBS), sodium citrate, laurilsarcosyl (ICN, USA); trypan blue stain, L-glutamine and 2-mercaptoethanol (Serva, Germany), penicillin and streptomycin (OAO "Biochimik" Saransk Russia); G-CSF (F.Hoffmann-La Roche Ltd, France); tabletted PBS ( mM">10 mM phosphate buffer+ 0,13 M NaCl + 2,7 mM KCl, pH 7,4), NPZ "EKO-servise" Russia.

Ph^+^ mononuclear cells used for the study were prepared from the peripheral blood (PB) or bone marrow (BM) of CML patients in chronic phase (CP), accelerated phase (AP), and blastic phase (BP) before or under treatment. Leucocytes and granulocytes are the main CML mononuclears content; namely, they have been investigated here. Characteristics of Ph+ cells and CML patients from whose PB and BM the mononuclears were isolated are given in Tables 1-3. In Ph+cells, bcr/abl RNA types as b3a2, b2a2, or e1a2 were assayed by RT-PCR (reverse transcription-polymerase chain reaction) according to [33, 35].

Ph^+^ mononuclear cells were isolated from 10-15 ml of PB or 1-2 ml of BM aspirate (from superior iliac crest) of CML patients at different phases of the disease. The heparinized (50 IU/ml) material was centrifuged for 30 min at 1500 rpm over Phycoll or Lymphoprep (1.077 or 1.119 g/sm^3^). The resulted light fraction was washed twice with PBS at pH 6.8 and once with a ≤-MEM media and then re-suspended in ≤-MEM media for analysis and cell cultivation. This fraction contained progenitor cells (blasts), lymphocytes, granulocytes, and monocytes, as well as some quantities of mature neutrophils, typical for CML mononuclear cells. The viable and dead cell number was counted 3 times in smears, stained by 0.2% trypan blue according to Romanovsky, with consequent cell counting in Goryaev's chamber.

Ph^+^ cells cultivation was performed according to [33]. Cell suspension of 2≤8·10^6^ cells/ml was incubated with ≤-MEM media containing 10-20% fetal calf serum (FCS, 2 mM L-glutamine, 10^-4 M 2-mercaptoethanol, 100 U/ml penicillin and 50 U/ml streptomycin, 25 mM HEPES-NaOH, pH 7.2-7.4 in 25 sm2^ plastic flask for 2-3 h) nonadherent cells were then centrifuged at 1500 rpm for 7 min and re-suspended with the same medium to 0.8≤1.4·10^6^ cells/ml and transferred to 24- or 96-well plates (12 wells per probe) and incubated at 37 ^o^C with 100% humidity and 5% CO2 without FCS for 2 h. Then 10-20% of FCS was added, and the cells were cultured for 6-14 days, selecting each sample from separate probes. Every point was tested in triplex. Ph^+^ cells in the collected samples were washed from FCS by centrifuging in ≤-MEM media and analyzed for their morphology, cellular composition, distribution in the cell cycle phases, and apoptosis. We had separately determined that 2-hour incubation of cells without FCS with consequent incubation with FCS diminishes cell proliferation during the next ~6 hours. After that, the proliferation rate is restored, and 12 hours later the cellular composition becomes the same as in the cultures with FCS.

The prepared probes were analyzed for the number of viable and dead cells and cell morphology in 3 zones of smear (100 cells in each) according to Romanovsky, the individual cell morphology was identified according to Abramov [34], and we also calculated the percentage of each cell type in every sample. We calculated cell concentration in samples in 10^6^ cells/ml and obtained plots for the kinetic curves of accumulation and depletion of different Ph^+^ cells and their subpopulations: proliferating cells (blasts, promyelocytes and myelocytes) - P and nondividing mature neutrophils: MM, B and S (metamyelocytes, band and segmented neutrophils) - D. The plots reflected the rate of production (or accumulation) of each differentiating cell type, transforming to the next subpopulation for this cell differentiation line. The mean error was ±5 ≤ 11%. We also studied apoptosis and cell cycle phase distribution by flow cytometry.

Ph-chromosome in PB and BM cells of our patients was identified cytogenetically (for 100 mitoses) or by FISH in the cytogenetical laboratory of the National Research Center for Hematology.

Flow cytometry for the analysis of the cell cycle phase distribution of the cultured Ph^+^cells selected during the incubation of Ph^+^ cells samples (5,000 cells each) after the isolation in Phycoll gradient were centrifuged for 7 min at 2000 rpm and 4^o^C, washed with PBS, and accurately fixed by cold 70% ethanol for 30 min at 4^o^C. Before measurement, the suspension was washed with PBS, centrifuged, and the pellet was incubated in 0.5 ml of PBS containing 5 mcg/ml of propidium iodide and 50 mcg/ml of ribonuclease A for 30 min at room temperature in a dark place [36, 37]. The cell measurements were done with flow cytometer EPICS-XL. Cells of granulocytes gate were analyzed by direct (FSC) and side (SSC) light scatter with simultaneous registration of the FL2 fluorescence by amplitude and impulse square (it allows to exclude aggregated cells, conglomerates, and debris) in the linear and logarithmic scales. Simultaneously apoptotic cells are detected. FL2-H particles with hypodiploid DNA located as a separate peak leftward of the peak of diploid cells (decrease of cell size not more than by 2 logs) were considered to be apoptotic. The percentage of apoptotic granulocytes was estimated within the granulocytic gate, where there is no cell debris. The DNA histograms from the same cell samples were analyzed for cell cycle phase distribution (S, G2/M). Its analysis was done with the help of previously developed specialized software. It was based upon an algorithm developed for asynchronous proliferating cell populations (SFIT-method) [38].

The kinetic plots of Ph^+^ leucocytes (number increase and death of Ph^+^ cells) were obtained using the percentage of viable and dead cells (see above) measured in 10^6^ cells/ml. The proliferation rate of leucocytes in whole and granulocytes in particular was estimated from the kinetic plots of its accumulation and depletion under cultivation and also from the sum of its subpopulations, defined by morphology. The kinetic curves showed that the accumulation and consumption of each studied type of cells parallels its transformation into the next subpopulations. The depletion of segmented neutrophils means its death.

Differentiation plots of Ph^+^ leucocytes and its subpopulations - lymphocytes, all myeloid cells, granulocytes, and its subpopulations, blasts, promyelocytes, myelocytes, metamyelocytes, band and segmented neutrophils - were defined, calculating the concentration of corresponding cells in samples and multiplying its fraction (defined by morphology in smears) by 10^6^/ml. The cell morphology in smears was assessed as already mentioned. The cellular composition was assessed in smears in 3 zones of 100 cells each. It should be noted that besides morphology Ph^+^ CML cells № 1.1 and № 2.6 were identified by CD antigens expression according to [39], where the results of identification and kinetics of cells according to both antigen expression and morphology are given and results are shown to be coincident [33, 39].

Kinetic plots of P/D efficiency. P/D of granulocytes in Ph^+^ cell cultures was defined as the P/D efficiency index that is the rate ratio of accumulation and depletion of proliferating cells (P cells sum) and neutrophils matured without dividing (D cells sum) under Ph^+^ mononuclear cells cultivation as defined above.

The P/D efficiency index was defined as the ratio of P cells (proliferation rate) and D cells (differentiation rate). It is equivalent to the ratio between the concentration of these cells, according to consideration V_P_ /V_D_ =K_P_ [P] t / K_D_ [D] t =K_P_/ K_D_ x[P] / [D], where V_P_ and V_D_ are the rates of P and D cells accumulation; K_P_ and K_D_, constants of rate; and [P] and [D], cell concentrations. K_P_/ K_D_ = K is the constant of relative P/D efficiency.

## RESULTS

In order to study the differences in PAmp;D in the Ph^+^ cell cultures of patients with different CML phases, we obtained kinetic plots for 34 samples of Ph^+^ mononuclear cells from the PB and BM of 23 CML patients under cultivation in strictly identical conditions. Ph^+^ mononuclear cells from PB and BM contain hematopoietic cells capable of self-renewal at PAmp;D and to PAmp;D for 2-3 cycles, forming the full set of Ph^+^ cells [33, 39].

The majority of mononuclear cells in CML are leucocytes [1, 7]; thus, we considered them as Ph^+^ cells. The leucocytes subpopulations include granulocytes, lymphocytes, monocytes; the granulocyte subpopulation contains myeloid precursors (blasts), promyelocytes, myelocytes and mature neutrophils: metamyelocytes (MM), band (B), and segmented (S) neutrophils.

**Fig. 1.**
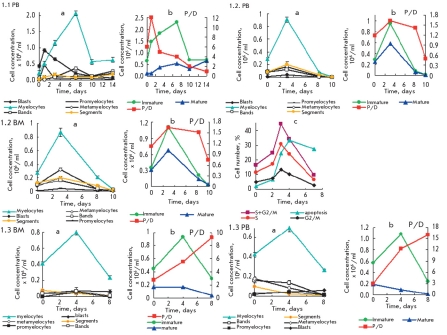
Kinetics plots of proliferation and differentiation (as accumulating and consuming) of type 1 Ph^+^ cells subpopulations in the suspension culture of mononuclear cells isolated from bone marrow (BM) or peripheral blood (PB) of chronic myeloid leukemia (CML) patients a - differentiation of granulocytes subpopulations with formation of proliferating myeloid precursor cells (blasts), promyelocytes, myelocytes, and neutrophils maturated without dividing: metamyelocytes, bands, and segmented; b - accumulation and consumption of immature proliferating cells, P cells, and neutrophiles matured without dividing, D cells, as well plots of proliferation and maturation efficiency as ratio of P and D cells accumulation rates - P/D index; c - plots of apoptosis and Ph^+^ granulocyte distribution in the cell cycle phases in culture. 1.1 PB - PB Ph^+^ cells from CML CP patient #1.1 before treatment, showing fast progression to CML BP. 1.2 PB and 1.2 BM - Ph^+^ cells from CML CP patient #1.2; 1.3 BM and 1.3 PB - Ph^+^ cells isolated from patient with CML AP under treatment (rapid progression to blastic phase). Samples characteristics, as well as proliferation and differentiation data of type 1 Ph^+^ cell, are shown in Table 1

**Fig. 2.**
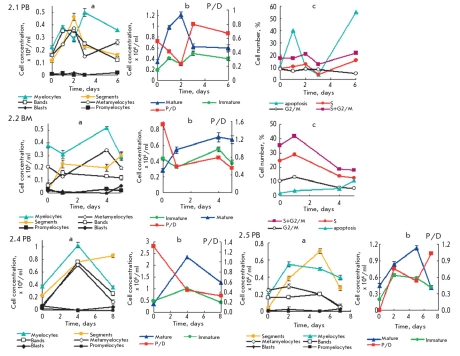
Kinetic plots of formation and transformation of type 2 Ph^+^ cells subpopulations as well as kinetics plots of their proliferation and maturation (as accumulating and consuming) in the suspension culture of mononuclears isolated from bone marrow (BM) or peripheral blood (PB) of chronic myeloid leukemia (CML) patients a-, b-, c- the same as in Fig. 1. 2.1 PB, 2.2 BM, 2.4 PB and 2.5 PB - samples of cells from corresponding CML CP patients. The prolonged observation didn't reveal signs of CML progression. Samples characteristics, as well as proliferation and maturation data of type 2 Ph^+^ cells, are shown in Table 1

**Fig. 3.**
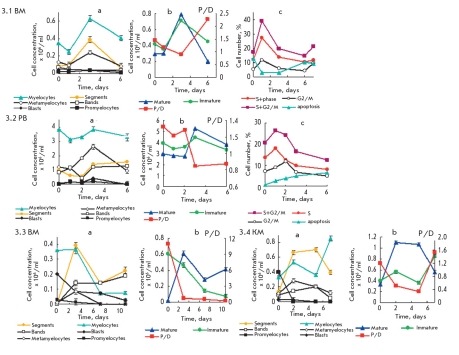
Kinetic plots of proliferation and maturation of type 3 Ph^+^cells with alternating efficient proliferation and efficient maturation stages according to 1/2 or 1/2/1 scheme in the suspension culture of mononuclear from BM or PB of CML patients a-, b-, c- the same as at Fig. 1. Ph^+^cell 3d type 3.1 BM, 3.2 PB, 3.2 BM, 3.3 BM and 3.4 BM - samples of cells from corresponding CML CP patients. Parameters characteristics of efficient proliferation and efficient maturation for this Ph^+^ cell type are shown in Tables 2 and 3

**Fig. 4.**
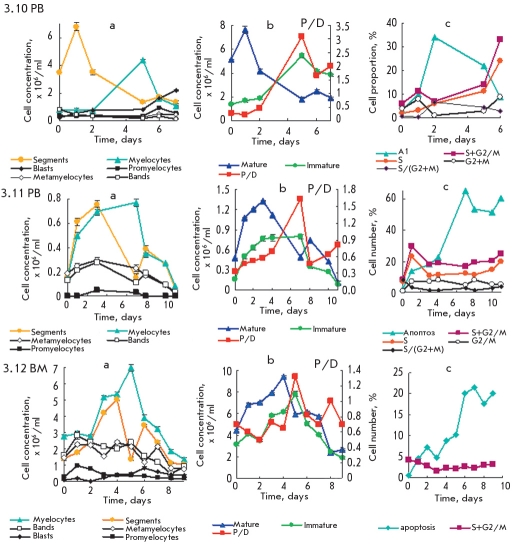
Kinetics plots of proliferation and maturation of subpopulations of 3d type Ph^+^cells that perform by alternating efficient proliferation and efficient maturation stages according to 2/1 or 2 /1/2 schemes in the suspension culture of mononuclears from BM or PB of CML patients. Parameters and characteristics of efficient proliferation and efficient maturation of this Ph^+^ cell type, are in table 2 and 3

The characteristics of Ph^+^ cells and corresponding CML patients are given in Tables 1-3. In cell samples obtained under cultivation, we obtained the kinetic plots of CML leucocytes proliferation and death and differentiation of Ph^+^ granulocytes subpopulations: blasts, promyelocytes, myelocytes, MM, B and S. In the same samples, we also assayed plots for apoptosis and distribution of Ph^+^ cells in the cell cycle phases (Fig. 1-4). The experiments showed a typical for CML elevated granulocytes content [1, 6, 14]. The shape of the plots indicates the rate of production or accumulation of the corresponding cell type, their transformation to next subpopulations, and final death. Thus, the resulting plots reflect the main differentiation processes (Fig.1-4 and Tables.1-3). The leucocytes differentiation coincides with known CML data [1-3, 21, 22, 40-42].

**Table 1:** Proliferation and differentiation of types 1 and 2 Ph+ cells in culture

N	Samples of mononuclear cells from PB or BM of CML patients			Proliferation and differentiation parameters					
	Sample number CML PB or BM	Diagnosis, treatment before sampling	Type of bcr/abl RNA, leucocytes ×10^9^/L; % blasts	P/D, [immature]/[mature]	P/D duration, days	[S]/[M]	S×10^6^ cells/ml, max/at day	Neutrophils × 10^6^ cells/mlmax /at day	Apoptosis(cell death) % /at day
1	2	3	4	5	6	7	8	9	10
Type 1 Ph+ cells, P/D1 ?1. Effective proliferation (accumulation rate of immature Ph+ cells is higher than neutrophils maturation rate). Concentration [immature] > [mature].									
1	1.1 PB	CP	b3a2 L115;3 %;	12-4 4-1	14	0,14-0,0-0,27	0,17	0,53/7-0,32-0,65/14	(28/7 50/14)
2 3	1.2 BM PB	CP, HU*	b3a2 L 72 blasts 3%	1,2-1,6 2,4-1	8 8	0,41 0,5	0,2 0,2/3	0,67 0,58/3	(33/4 30/9
4 5	1.3 KM PB	AP Chem**	b2a2 blasts 17%	3-9 1-13	> 8	0,1-0,54 0,1-0,31	0,13/8	0,17/0-4	(14/8)
6	1.4Blood	lymphoidBP,Chem**	b2a2 blasts 30%	>20- 2	> 6	0,0	0/0-6	0,4/6	(47/6 )
7	1.5 PB	CP,HU*	b3a2 L 175 Blasts 5%	1,2-2,5	< 8	0,14-0,53	0,08/4	0,32-0,2/4,8	(21/7)
Type 2 Ph+ cells, P/D2 ≤ 1. Effective maturation ( neutrophils maturation rate is higher than accumulation rate of immature Ph+ cells). Concentration [mature] > [immature].									
1	2.1 PB	CP>BP HU*	b3a2, L 188-145, 2 %	0,3-0,8	> 6	1,0-1,62-0.4	0,47/2	1,2/2	40/1 4/3 55/6
2	2.2 BM	CP HU*4 days leter	b3a2	0,8-1,5	> 5	0,8-0,4-1,0	0,5/2	0,7/1 0,6/5	(4/1 11/5)
3	2.3 PB	CP	b3a2	0,4-1,0	~ 6	0,4-0,8	0,25/ 3	0,7/3	(2/3 19/7)
4	2.4 PB	CP, 2 risk group, ***	b3a2,L 165,blasts 2%	0,3-1, 2	~ 7	0,73/4 2,4/8	0,86/ 8	2,4/4 1,3/8	(2/4 33/11)
5	2.5 PB	CP	b2a2	0,1-1,1-1,6	~ 7	1,4/5	0,7/5	1,13/5	(3/2 2/5 17/7)
6 7	2.6 PB BM	CP	b3a2	0,2-1,00,3 -1,0	>9	8/6 24/11	0,41/6	0,7/6 0,1/11	(2/6 24/11)
8 9	2.7 BM PB	CP	b2a2	1,5 1,5	~ 4-6	--	--	1,1/3 1,3/7	(15/1 2/2-7 2/3 12/8)

The treatment before sampling: *hydroxyurea; **chemotherapy. *** interferon CP, AP and BP - chronic phase, accelerated phase, and blastic phase - CML phases.

**Table 2:** Parameters of alternation for efficient proliferation and efficient maturation stages under condition of proliferation and

Item №, Figure №	№ XML (*)	Parameters of proliferation stage Max or interval (days)				Parameters of maturation stage Max or interval (days)				[S]/[myelocytes]	Apoptosis(death)%/at day
		P/D1	10^6^ cells/ml			P/D2 Min	Max, 10^6^ cells/ml				
		[immature]	[mature]	[S]	[immature]	[mature]	[S]				
Alternation stages according to 1/2 or 1/2/1schemes with changing accumulation rates and Ph+cell concentration [immature] >[mature] > [mature] > [immature] > [immature] > [mature]											
1, Fig. 3	3.1 BM (1/2/1)	1,4-1,1 1,2-2,3	0,35-0,5 0,64-0,5	0,29-0,5 0,63-0,2	0,1-0,25 0,3-0,1	0,9 (at 3 day)	0,71 (at 3 day)	0,8 (at 3 day)	0,4 (at 3 day)	0,5-0,2	5-12
2,Fig.3	3.2** PB (1/2)	1,3-1,2	0,4	0,30	0,05	0,85	0,4-0,3	0,53	0,16	0,5-0,25	< 5
3 4 Fig.3	3.3 BM PB (1/2)	11-2 1,32-1,5	0,6-0,46 0,5-0,8	0,02-0,5 0,4-0,5	0,01-0,4 0,14 -0,25	0,1 1,0-0,2	0,5-0,1 0,5-0,1	0,6 (3 day) 0,6 (7 day)	0,4-0,2 0,3-0,2	0,1-2,5 0,1-1-2,5	(7 at 11 day)
5 Fig.3	3.4 BM (1/2/1)	1,2 1,53	0,4-0,8-0,5-1,1	0,34	0,17	0,52-0,34	0,57-0,34	1,1	0,7	0,4-1,3-2	(18 at 5 day)
6	3.5BM (1/2)	3,43-1,0	1,95	1,65	0,03-0,37	1,0-0,3	1,53-0,4	1,53-1,21	0.37-0,5	0,19-0,25-2,0	(30 at 11day)
7	3.6BM(1/2)	1,61	0,61	0,52	0,24	0,28	0,13-0,56	0,52-0,4-0,6	0,24	0,13-0,5-1,0	(22 at 11 day)
8 9	3.7BM (1/2)PB(1/2/1)	2,5-1,4 2,2-1,2 1,2-1,4	0.65-0,60 0,51-0,54 0,38-0,32	0,55 0,5 -0,54 0,4-0,2	0,3 0,05-0,19 0,15-0,07	0,7-0,4 0,78	0,55 0,57-0,38	0,83 0,73	0,53 0,27	0,1-0,5	(30 -35 at 10-11 day)
10	3.8 BM (1/2)	3,9-1,5	0,53-0,35	0,14-0,35	0,02-0,22	1,5-0,5	0,35-0,23	0,35-0,44	0,2-0,4	0,04-0,3	(45 at 7 day)
Alternation stages according to 2/1 or 2/1/2 schemes with changing accumulation rates and Ph+ cell concentration: [mature] > [immature] > [immature] > [mature]> [mature ] > [immature]											
11, Fig.4	3.10*PB(2/1)↔	1,4-3,1-2,0	0,3-0,6-0,4	0,3-0,2	0,17-0,25	0,2-0,5	0,13-0,3	0,5-0,8-0,3	0,35-0,7	8,5-0,3	34-22 at 2-5 days
12, Fig.4	3.11PB(2/1/2)↔	1,15-1,63	0,8-0,6	0,6-0,8-0,1	0,43-0,3	0,33-1,150,75-0,8	0,2-0,8,0,6-0.8-0,1	0,5-1,3-0,8,0,8-0,1	0,16-0,75-0,4,0,39-0,06	1,2-0,2	35 at 1 day,63 at 4 days
13,Fig.4	3.12 BM (2/1/2/1/2)↔	1,0-1,32 1,0	0,7-0,78 0,24	0,6 0,24	0,14-0,35 0,12	0,5-0,7-1,0 1,0-0,8-1,0	0,6-0,7 0,6 0,2	0,4--0,95 0,6	0,14-0,5 0,34 0,1	0,9-0,2	7, 22 at 6, 11 days
14	3.13 PB (2/1)↔	1,7-2,5	0,39	0,4-0,2	0,2-0,1	0,45-1,7	0,42	0,9-0.4	0,3-0,2	0,9-1-9	(48 at 8 days)
15	3.14BM(2/1)↔	1,0-1,34	0,6-0,5	0,4	0,4-0,2	1,1-0,7	0,4-1,0	0,4-1,37	0,12-0,92	0,3-1,0	(45 at 5 days)

Footnote: *alternation scheme; **acceleration phase; BM - bone marrow, PB- peripheral blood; brackets [ ] show cell concentration; hyphen - interval of values on the kinetic plots; Figure ↔ shows that 1st stages data for alternation 2/1 and 2/1/2/1 are given earlier than that of 2d stages according to the subtitle of the table.

**Table 3:** The characteristics of crossings at alternation of proliferation and maturation stages during P&D of type 3 Ph+ cells in culture. See notes to Table 2

N Fig.	N CML patient	Stage alternation scheme	Crossings of accumulation rate plots for proliferating [immature] (stage 1) and maturating cells [mature] (stage 2)							Cell concentration in crossings, 106 cells/ml			
			Time of crossing, Day		Stages duration, days		P/D indices in crossing points N			Mature and immature		Segmented (S)	
			1	2	1	2	1	2	3	1	2	1	2
1 Fig.3	3.1 M	1/2/1	2	3,5	2 2.5	1,5	1,1	1,2	-	0,5	0,65	0,24	0,34
2 Fig.3	3.2 PB	1/2	2,5	-	2,5	>4,5	1,06	-	-	0,4	-	0,1	-
3 4	3.3 BM PB	1/2 1/2	2,5	5,5	2,5 5,5	8,5 5,5	~ 2 1,0	- -	- -	0,49 0,53	- -	0,26 0,38	- -
5 Fig.3	3.4 BM	1/2/1	0,2	6	~ 0,2	5,8	1,15	1,2 -		0,37	0,8	0,17	0,58
6	3.5 BM	1/2	5	-	5	3	1,0	-	-	1,53	-	0.37	-
7	3.6 BM	1/2	4,5	-	4,5	3,5	1,06	-	-	0,53	-	0,24	-
8 9	3.7 BM PB	1/2 1/2/1	2,5 2,5-8,5	2,5 2,5	8,5 8,5	1,5 1,2	- 1,25	- -	0,53 0,54	- 0,4	0,4 0,17	- 0,15
10	3.8 BM	1/2	5	-	5	>2	1,5	-	-	0,35	-	0,15	-
11 Fig.4	3.10 PB	2/1-	3	-	4	3	1,4	-	-	0,33	-	0,3	-
12 Fig.4	3.11 PB	2/1/2/1-	5,5	7,5 11	2 0	5,5 3,5	1,15	1,15	0,80	0,75 0,1	0,6	0,43 0,06	0,3
13 Fig.4	3.12 BM	2/1/2/1/2-	4,5	6 0 8	1,5 4,5	0 2,5 1	1,05 1,0	1,0	1,0	0,62 0,24	0,72 0,24	0,13 0,34	0,35 0,11
14	3.13 PB	2/1-	4,0		1 4		1,7	-	-	0,4		0,2
15	3.14 BM	2/1-	4,0		1 4		1,0	-	-	0,6		0,4	

The mean value of P/D efficiency index at crossings of proliferation and maturation rate plots at alternation stages 1 and 2 is 1.06 ± 0.23 (21,7%). BM - bone marrow, PB- peripheral blood; brackets [ ] show cell concentration; hyphen - interval of values on the kinetic plots; Figure - shows that 1st stages data for alternation 2/1 and 2/1/2/1 are given earlier than that of 2d stages according to the subtitle of the table.

Besides the differentiation rate of separate subpopulations (Fig.1-4 a), we studied the plots of overall proliferation and maturation rate. In the 1^st^ case, these were the plots of accumulation of P cells differentiating in parallel with proliferation and including blasts - myeloid cell precursors, promyelocytes, and myelocytes. In the 2^nd^ case, they were the plots of accumulation of neutrophils - D cells: the sum of MM, B, and S matured without dividing (Fig.1-4 b).

## TYPES OF CML CELLS

Myelopoiesis (PAmp;D of myeloid division of hematopoiesis) begins with the proliferation and differentiation of hematopoietic progenitor cells and their immature progenies. Then PAmp;D continues with neutrophils maturation without dividing [3, 40, 41]. In this study, we obtained and analyzed ex vivo PAmp;D kinetic plots in 34 samples of Ph^+^ cells from the PB and BM of 23 CML patients. They demonstrated evident differences in the PAmp;D of Ph^+^ cells from individual CML patients.

The kinetic plots of proliferation and maturation reflect the rate of two crucial PAmp;D processes: proliferation with differentiation and differentiation without dividing (maturation of neutrophils). The rate ratio of proliferation (P cells accumulation) and maturation (D cells) reflects the efficiency of Ph^+^ cells proliferation relative to its maturation without dividing and is expressed in the P/D index, an indicator of P/D efficiency. One can see from the kinetic P and D cell plots that the proliferation rate can be higher or lower than that of maturation; thus, PAmp;D can give advantage to proliferation (effective proliferation) or to maturation (effective maturation). In the first case, the P/D efficiency index P/D^1^≤1; in the second, P/D^2^ ≤ 1 (Fig.1-4C, Tables 1-3).

In 20% of CML patients, P&D is associated with continuous effective proliferation only. These are type 1 Ph^+^ cells, their P/D^1^ =1-20 (Fig. 1, Table 1). PAmp;D of Ph^+^ cells from other CML patients is associated with effective maturation only with P/D^2^ ≤ 1. It corresponds to type 2 Ph^+^ cells (~30% of cases) (Fig. 2 b, Table 1). During PAmp;D, these indices vary in their limits of PAmp;D. The Figures 1, 2 andTable 1show that the continuance of PAmp;D of Ph^+^ cells types 1 and 2 (7-14 days) is comparable with that for 1-2 cell cycles of hematopoietic cell clon [3, 40, 41].

The PAmp;Ds of most samples of Ph^+^ cells are characterized by repeated alternations of efficient proliferation and efficient maturation with the prevalence of the proliferation rate and high P/D index (P/D^1^≤ 1-4), or with the prevalence of the maturation rate and low P/D index (P/D ≤ 1) (Fig. 3, b, Fig. 4, b and Tables 2, 3). These Ph^+^ cells constitute type 3 of Ph^+^ cells. The alternations of proliferation (1^st^ stage) and maturation (2^nd^ stage) are clearly identified by crossings of the kinetic plots of accumulation of proliferating (P [immature]) and matureting (D [mature]) cells (Figs.3 and 4b, Table 3). Proliferation and maturation can be accompanied by 1-3 such alternations that are changing each other according to the following schemes: 1/2 -1/2/1 or 2/1-2/1/2, with frequency from 1 to 5 days, rarely 0.2-6 days (Tables 3 and 4). In alternation according to schemes 1/2 - 1/2/1, the rates and, hence, cell concentrations, are changing in the following series: [immature]> [mature]> [mature]> [immature]> [immature]> [mature] (Figs.3: 3.1- 3.4, b). In alternation according to schemes 2/1 - 2/1/2, the rates and, hence, cell concentrations, are changing in the following series: [mature]> [immature]> [immature]> [mature]> [mature]> [immature] (Figs.4: 3.10-3.12 ,b). PAmp;D of Ph^+^ cells type 1 and 2 can be considered as a particular case of prolonged alternation of proliferation or maturation of Ph^+^ cells type 3.

At the moment of crossing of effective proliferation and effective maturation plots, the alternation of stages, rate of Ph^+^ cells accumulation, and other PAmp;D parameters takes place. At the moment of crossing, the proliferation and maturation rates, as well as the indices of their efficiency, become equal. At that moment, P/D = 1.06±0.23 regardless of scheme, time point, alternation sequence or cell concentration (Tables 2 and 3). These moments can be called critical or equilibrium points. Other characteristics that may be expected to be equal (concentrations of immature cells, mature neutrophils (0.4-0.7x10^6^/ml) and segmented neutrophils only (0.1-0.4x10^6^/ml)) in fact were similar (Table 3). However, the prominent variations (1.53-0.1x10^6^/ml for BM 3.5, PB 3.10, 3.11 and BM 3.12 in Table 3) suggest the concentration's dependence.

During alternations, the cell characteristics are continuously changing (Table 2). The parameters increase or decrease, passing maximums and minimums. These events are usually asynchronous (Fig. 1-4 and Table 2). The pattern of P/D indices changing depends upon the stage. They vary within the limits P/D^1^ =1-4, P/D^2^ ≤1. The only exception was BM № 3.3, where P/D^1^ decreased from 11 to 1 3 days before it reaches a steady-state and the maturation stage begins. Under maturation, stage P/D^2^ varies from 0.1 to 1, untill a new proliferation stage with P/D^1^>1. It means that the rates of proliferation and maturation - their P/D efficiency indices and concentrations of proliferating and maturating cells (especially segmented neutrophils) - are rather significant parameters of Ph^+^ cells PAmp;D.

Analysis of the kinetics of granulocytes subpopulations development, the kinetics of apoptosis, and distribution of Ph^+^ cells in the cell cycle phases in combination with differences in P/D index and the rates of proliferation and differentiation stages of Ph^+^ cells PAmp;D revealed the following.

Proliferation and differentiation of type 1 Ph^+^ cells prolonged effective proliferation with P/D index>1-2 ÷ 20 and amount of Ph^+^ cells in S+G2/M phases ≤ 20-45%; the proliferation rate prevails over maturation (Figs.1.2, 1.3.a ,b, c). PAmp;D is associated with enhanced accumulation of myelocytes, promyelocytes, and/or blasts and low accumulation of neutrophils, maturing without dividing (Figs.1, a, b,: 1.1-1.3 and 1.4 in Table 1) and with a high level of apoptosis (Fig.1, c). The lower neutrophils content characterizes the higher P/D index (Fig.1: 1.1-1.3, b) and CML progression (Table 1). These Ph^+^ cells were isolated from the PB and BM of CML patients at all phases of the disease during progression and amounts to one-fifth of all studied samples of Ph^+^ cells.

Proliferation and differentiation of type 2 Ph^+^ cells is characterized by low efficiency (index P/D ≤ 1) with the prevalence of the maturation rate over the proliferation rate and apoptosis blocking. PAmp;D of type 2 Ph^+^ cells is associated with prominent neutrophils accumulation inhibiting the Ph^+^ cells proliferation (Fig.2, Table 1). Apoptosis inhibition during neutrophils maturation is not synchronous to myelocytes accumulation and induction of apoptosis. The decrease of the P/D index is associated with neutrophiles accumulation; the increase of P/D, with depletion of segmented neutrophils (Figs.2, a b, c; 2.1 - 2.7, Table 1). The mature neutrophils concentration is significantly higher than that of proliferating Ph^+^ cells.

An important characteristic of proliferation is the distribution of cells in the cell cycle phases. The kinetic plots of type 2 Ph^+^ granulocytes distribution in the S and G2/M phases show that their changing during proliferation and maturation depends upon their source - CML patient. Figure .2.1, 2.2, c shows that the portion of proliferating Ph^+^ cells in phases S+G2/M ≤ 20-40 %; and in G2/M phase, ~ 15%. Another proliferative index - S/(G2+M) ratio is 1.2. The maximum percentage of cells in S+G2/M at day 1 was 43 %; and in S phase, ~ 30%, rapidly declining to 12%. P/D index at the same time is 0.6-0.8 (Fig. 2.2 b). The percentage of proliferating types 1 and 2 Ph^+^ cells is similar (Fig. 2.2 c and Fig. 1.2 c). But the duration of elevated percentage of cells in S+G2/M phases in type 2 Ph^+^ cells is shorter than in type 1, where the portion of proliferating cells ≤43% is maintained for nearly 6 days with the S/(G2+M) ratio ~2.4-2.7 and P/D efficiency 1.2-1.6. In terms of PAmp;D of type 2 Ph^+^ cells, that means the prevalence of maturation over proliferation. Under cultivation, the percentage of proliferating malignant K562 cells (derivate of CML blastic phase) in S+G2/M phases reaches 44±3% with little changes in the next 7 days. The S/(G2+M) ratio at that time reaches 4.4. For comparison with these values, the PAmp;D of type 2 Ph^+^ cells is characterized by a decreased percentage of cells in S+G2/M phases (<20 - 40%) with a shortened duration of proliferation (<3 days) and low P/D efficiency ≤ 1 (Figs.2.1 and 2.2, b, c).

The kinetic plots of type 2 Ph^+^ leucocytes differentiation show ~80% granulocytes content, while the percentage of myelocytes is not more than 25%. The percentage of mature neutrophils, especially segmented, reaches ~40% (Figs.2.1-2.5, b and 2.1-2.6 in Table 1). During PAmp;D, all neutrophils are actively accumulated with little consumption. The maximal concentration of segmented neutrophils at times is twice that of myelocytes (Figs 2.1-2.5 a, b, c, and Table 1), emphasizing the role of mature cells (especially segmented neutrophils) in the regulation of PAmp;D of type 2 Ph^+^ cells.

In summary, the population of type 2 Ph^+^ leucocytes increases by 2-4 times under low P/D efficiency and a proliferation rate lower than that of maturation. It is associated with the accumulation of neutrophils (especially segmented), the decrease of the myelocyte content, and apoptosis inhibition. It means that the excessive accumulation of neutrophils (due to apoptosis blockage) inhibits the proliferation of their own precursors and the differentiation of neutrophils (metamyelocytes, band and segmented), probably through a feedback mechanism.

The ratio [S]/[M] is usually a small value; during proliferation and differentiation of type 2 cells, it increases, exceeds 1, and reflects the accumulation of segmented neutrophils, synchronous to the decrease of myelocytes accumulation. The neutrophils accumulation reaches a maximum at day 2 or 4 (Fig.2: a, b) that corresponds to a minimum accumulation of myelocytes and low P/D index. Figure 2 a, b shows the increase of the P/D index because of D cells concentration decrease at day 4.

The content of type 2 Ph^+^ cells is one-third of all studied samples; all of them isolated from CML chronic phase (CP) patients. The clinical observation of CML patients with type 2 PAmp;D shows prolonged life duration in case of successful therapy.

Proliferation and differentiation of type 3 Ph^+^ cells takes place as repeated alternation of proliferation (stage 1) with maturation (stage 2) and changing of the P/D efficiency index from P/D^1^> 1 to P/D^2^ ≤ 1 etc. (Tables 2 and 3). At the first stages of alternation, the myelocytes accumulation under activ apoptosis prevails over the accumulation of maturating neutrophils. Second stages of alternation are characterized by efficient maturation with apoptosis blockage and accumulation of enhanced concentration of neutrophils that inhibits further proliferation (Fig. 3, 4 and Tables 2 and 3). It is accompanied by an inversion of the sequence of neutrophils accumulation, as in the PAmp;D of type 2 Ph^+^ cells (Figs. 2-4 a, b, c) considered below.

The transition to the next alternation stage 1 according to schemes 1/2/1 and 2/1/2 is associated with apoptosis induction, depletion of segmented neutrophils, and accumulation of immature Ph^+^ cells. Thus, at the crossing of the proliferation and maturation plots, the effective proliferation changes to effective maturation and vice versa; the characteristics and parameters of the same (but already passed) stage are restored.

The P/D efficiency indices depend upon the alternation stage, as discussed above (Tables 2 and 3). At the first stages, index P/D^1^>1; at next stages, P/D^2^ ≤1.

The alternation of the highly effective proliferation of Ph^+^ cells and relatively ineffective neutrophils maturation results in increasing and decreasing P/D efficiency (Table 3 and Figs. 3 and 4). It allows to maintain a moderate efficiency of PAmp;D on the whole. It corresponds to alternating changing of type 3 Ph^+^ cells parameters (Tables 2 and 3). Taken together, these results mean alternation between the efficient proliferation and efficient maturation of the participation of segmented and other mature neutrophils in the regulation of type 3 Ph^+^ cells PAmp;D through inhibition of Ph^+^ cells proliferation at the maturation stage. A similar regulation can be seen during PAmp;D of type 2 Ph^+^ cells.

Alternating efficient proliferation and efficient maturation according to 1/2 scheme, beginning from stage 1 (Fig. 3.6, Table 2), have PAmp;D characteristics compatible to type 1 Ph^+^ cells. The content of myelocytes and proliferating cells in S+G2/M phases increases,> 30-45%, at days 1-2 (Fig. 3), while the neutrophils content drops. At stage 2 of alternation, the segmented neutrophils concentration increases, the P/D^2^ index drops ≤ 1, the myelocytes content drops, and the percentage of Ph^+^ cells in S+G2/M phases becomes ≤25%. At the beginning of the next stage 1 - the proliferation stage that happened at day 7 - the percentage of cells in the S+G2/M phases again begins to increase (Fig. 3.1 c) and other parameters of stage 1 restore. The alternation of stages 1 and 2 is analogous to the PAmp;D of Ph^+^ cells types 1 and 2, correspondingly, but their duration is shorter. The overall duration of stage 1 of Ph^+^ cells type 3 is 4-5 days - 3 times as much as in its stage 2 (Table 3).

The second stage of alternation of the PAmp;D of type 3 Ph^+^ cells - Ph^+^ cells corresponds to P/D^2^≤1 values, high segmented neutrophils concentrations, apoptosis block, and minimal concentration of myelocytes (Fig. 4 a, b, c and № 3.10-3.14 in Table 2). Under apoptosis induction and decrease of segmented neutrophils concentration, accumulation of myelocytes and the P/D^1^ index again begin to increase. The maximums of P/D^1^ and myelocytes accumulation coincide with minimum segmented neutrophils concentration at day 5. The mature neutrophils accumulation leads to a drop in P/D^1^, decrease of myelocytes concentration, and establishment of the efficient maturation stage. These properties are coherent with the characteristics of the PAmp;D of type 2 Ph^+^ cells and other patient cells with PAmp;D type 3, according to the 2/1/2 alternation scheme (Fig. 4 a,b,c; other samples data in Table 2).

Inhibition of apoptosis and myelocytes proliferation by segmented neutrophils is clearly seen in Figures 3 and 4 a, b, c, etc. in Table 2. At the same time, the more active the Ph^+^ cells proliferation and apoptosis, the higher is the P/D^1^ index (for example, Fig.3 a, b, c, and Table 2).

The distinctive feature of type 3 Ph^+^ cells PAmp;D according to the 2/1/2 scheme, beginning with stage 2, is in a prompt and significant accumulation of segmented neutrophils at the beginning of PAmp;D and in prominent apoptosis blocking, especially Ph^+^ cells proliferation inhibition and distortion of maturation. The apoptosis rate reaches a maximum at 2-5 days and equals ~35% (Fig.4, a-c), corresponding by PAmp;D time to the depletion and complete death of segmented neutrophils. At the beginning, maximum cell proliferation at the maturation stage is due to the accumulation and depletion of segmented neutrophils; the second maximum of Ph^+^ cells proliferation is already observed due to transition to the efficient proliferation stage after the death of segmented neutrophils (Fig.4: 3.10, c).

The percentage of cells in particular cell cycle phases for 2/1 and 2/1/2 alternations is also linked to segmented neutrophils accumulation and corresponds to a decreased cell number in S+G2/M phases. In Figure 4, a-c, one can see maximums at day 1 ~10% (Fig.4: 3.10 BM), ~25 % (3.11 PB), and less than 5% (3.12 BM). At days 2-10, the portion of this pool significantly decreases. Under the next short proliferation stage at days 10-11, it slightly increases with a simultaneous increase of the P/D^1^ index. Taken together with the decrease of P/D^2,^ it means the inhibition of Ph^+^ cells proliferation by neutrophils, more prominent with a more frequent alternation of prolonged maturation stages.

Figures 4: 3.12, a-c indicate that under alternation of 2/1/2/1 stages inhibition of proliferation proceeds with P/D^2^ = 0.1-0.9. Under these alternations, the cell number in the S+G2/M phases is 5 % lower for eleven days. At low proliferation at the maturation stage, the apoptosis rate is less than 10%; at days 6-11, it increases only to 20% and only at the transition to stage 1. Overall, the time Ph^+^ cells № 3.12 stay under the effective maturation stage is three times longer than at stage 1. It leads to the most prominent inhibition of proliferation and apoptosis in type 3 Ph^+^ cells. In Figures 4: 3.11 a, b, c, maximum apoptosis corresponds to minimum segmented neutrophils accumulation, maximum myelocytes accumulation, and P/D index rise. Type 3 of PAmp;D was found in ~50% of the Ph^+^ cells studied (all from CML chronic phase patients). Among them, 2/3 constitute Ph^+^ cells with alternation of 1/2 and 1/2/1 stages.

Apoptosis block segmented neutrophils accumulation and inhibition of Ph^+^ cells types 2 and 3 proliferation by neutrophils during effective maturation. The maturating neutrophils differentiate in the sequence M>MM>B>S with consequent apoptosis of segmented neutrophils. The accumulation of maturating neutrophils decreases in the order S>B>MM [3]. If the transport of neutrophils to other tissues is impossible, they should die by apoptosis; therefore, the observed prominent S accumulation means the block of apoptosis.

The maturation of Ph^+^ cells type 2 and at 2^nd^ alternation stages of Ph^+^ cells type 3 is associated with apoptosis blockage, elevation of mature neutrophils concentration, inhibition of myelocytes accumulation and decrease of the percentage of cells in S+G2/M and/or shortening of their staying in those phases. These events explain the inhibition of proliferation by neutrophils with blocking of apoptosis. It should be noted that apoptosis induction is usually observed under depletion of cytokines in cultural media [16-18, 25, 26, 29, 43-45]. During maturation stages of PAmp;D for Ph^+^ cell, apoptosis is blocked without addition of cytokines in cultural media.

At S concentration ~2-3x10^5^ cells/ml and high S+G2/M cells content, the apoptosis rate is low (<5-10%); at day 5, it hardly reaches 10% (Fig. 2.2 a,c). Cell death in other cases (by trypane blue staining) is also inhibited. In cases 2.2-2.4 BM and 2.6 PB, the apoptosis level didn't exceed 10-20% (Table 1). As one can see in Figure 2.1 a, c, at day 1 of proliferation, the maximum apoptosis level reached 40% and 60% at day 6 with synchronous increase of myelocytes accumulation. It means that inhibition of myelocytes accumulation depends upon apoptosis blocking not directly but is rather mediated by the neutrophils accumulating due to the lack of apoptosis.

During PAmp;D of type 2 Ph^+^ cells, segmented neutrophils are even capable of accumulating with a separate peak at days 2-6 (with [S]> 0,2-0,3x10^6^/ml), as can be seen in Figures 2 a, b. It corresponds to a low apoptosis level and minimum myelocytes accumulation (Figs. 2.1 b, c). The myelocytes accumulation plot is broken for the period of significant S accumulation (with a maximum at day 2), while the P/D index decreases. Myelocytic proliferation is inhibited until the segmented neutrophils concentration decreases due to apoptosis. After the death of a significant part of S, the pattern of myelocytes accumulation restores and the P/D index increases (Figs. 2.1 b), but it remains ≤1. The maximum of S accumulation (Figs. 2.1 a, b, c) at days 1 and 5 also corresponds to the minimum of myelocytes accumulation, lowering of P/D, and activation of apoptosis. A similar S accumulation (at days 2-5) inhibits myelocytes accumulation and decreases P/D, as can be seen in Figures 2.5 a, b. The death of S at day 5 restores the increase of myelocytes concentration and P/D value. Figures 2.2 a, b show that maximum S accumulation corresponds to minimum accumulation of myelocytes; i.e., maximum inhibition of myelocytes accumulation. At the same time, maximums of myelocytes accumulation and apoptotic activity in Figures 2.1 a, b, c coincide. The same rule applies during PAmp;D of type 3 Ph^+^ cells.

Thus, the low PAmp;D efficiency of Ph^+^ cells type 2 and type 3 at stages of effective maturation leads to prominent neutrophils accumulation, decrease of the proliferation rate, in comparison with the maturation rate, and decrease of of Ph^+^ cells proliferation. Inhibition of Ph^+^ cells proliferation by neutrophils takes place under alternation of all stages of effective maturation and means the participation of neutrophils in the regulation of Ph^+^ cells PAmp;D.

The regulation of PAmp;D Ph^+^ cells types 2 and 3 consists of interdependent synchronous and asynchronous processes. Apoptosis inhibition with accumulation of neutrophils, maturing without dividing, proceeds asynchronously to myelocytes accumulation, apoptosis induction, and increasing efficiency. Myelocytes accumulation is synchronous with apoptosis induction, proliferation activation, and increase of P/D efficiency.

Inversion of mature neutrophils accumulation order. Neutrophils maturing without dividing (D cells) by definition differentiates in the order M>MM>B>S with consequent apoptosis of S [3, 40, 41, 45]. Under an equal differentiation rate, it can be estimated to have the same order of accumulation. In some cases - at PAmp;D of type 1 Ph^+^ cells and effective proliferation stages in type 3 Ph^+^ cells - this order is actually observed.

During alternation at maturation stages of Ph^+^ cells types 2 and 3, maximum and minimum values in the kinetic plots of neutrophils accumulation point to a change of their accumulation order (Figs. 2, 3 and 4, a). It means that the accumulation rate of S, B and MM changes nonuniformly, and that their accumulation order is inverted. At that time, the accumulation of myelocytes decreases adjacent to the corresponding neutrophils peaks. Moreover, the order of neutrophils accumulation from the same Ph^+^ cells inverts during the maturation process, restoring after the transition to proliferation stages.

Figure 2, a shows that order changes with increase or decrease of the neutrophils accumulation rate are frequent. It can be illustrated by the crossings of the neutrophils accumulation order plots with the inversion of their direction. For example, the order M>B>MM~S, visible at day 1, consequentially inverts to S>MM~B>M at day 2, to M>B~S>MM at day 3, and to M>MM>S>B at day 6. In this figure the equal neutrophils accumulation rates after the crossing of plots at days 4-5 leads to an almost normal neutrophils accumulation order - M>>MM>S≤B. In Figure 2:2.1, b one can see the usual accumulation order - M>MM>B>S - at day 0, but the inversion of this order leads to M>S>B~MM at day 1, then to M>>MM>S>B at day 4, and later to S accumulation with depletion of M, MM, and B. In Figure 2:2.4, a nearly equal accumulation rates of 3^rd^ neutrophils at day 4 (M>B~S~MM) and at day 8 result in S>>M>B>MM. In Figure 2:2.5, a, accumulation order M>S>MM>B at day 2 transforms into S>M>>MM~B at day 5.

These results mean that the inversions of accumulation order, i.e. accumulation rate of neutrophils as a whole and their fractions, are frequent. The restoration of the usual neutrophils accumulation order corresponds to the increase of myelocytes content and P/D efficiency index and promotes the transition to effective proliferation. Rate change and inversion of neutrophils accumulation order, synchronous to the decrease of P/D efficiency index (Figs. 1 and 4, d; 2, 3 and 4, b), points to the direct participation of neutrophils in the PAmp;D regulation of Ph^+^ cells types 2 and 3 with inhibition of their proliferation.

Under maturation stage of Ph^+^ cells type 3 with alternation according to schemes 1/2, 1/2/1 in Figure 3, a the inversion of the accumulation order manifests as transformation from M>MM>S>B to M>S>MM>B and then again to M>MM>S~B. At alternations according to schemes 2/1 and 2/1/2, the inversion of neutrophils accumulation order is seen in Figure 4, a. The neutrophils concentrations are decreasing in row [S]>>[M]>[B]>[MM], as well as P/D index (Fig. 4, b).

Thus, there is inversion of order for consequent neutrophils maturation with their maximum neutrophils accumulation, whose initial order of neutrophils maturation is able to restore by transition to the alternation of the efficient proliferation stage. The extent of this inversion and restoration of the neutrophils accumulation order depends upon the duration of maturation and proliferation stages. Inversion of the accumulation order of M, MM, B, and S is another interesting property of Ph+ cells types 2 and 3 in culture. The rate and extent of neutrophils accumulation is changed: at first it is S; then, B and MM.

Under effective maturation, the neutrophils accumulate in significant quantities due to apoptosis blocking (Fig. 2). They participate in the regulation of Ph^+^ cells types 2 and 3 PAmp;D not only by inhibiting Ph^+^ cells proliferation, but also by inhibiting neutrophils maturation. Inhibition of neutrophils differentiation in Ph^+^ cells types 2 and 3 is revealed at inversion of the initial neutrophils accumulation order with the increase in their concentration (Fig. 2, 3 and Fig. 4, also in Tables 1-3). Inversion of the initial neutrophils accumulation order leads to gradual change of the neutrophils accumulation sequence M>MM>B>S to S>B>MM>M, and the increase of their concentration in the same order is assumed to proceed by a feedback mechanism. The present inversion of the accumulation order and increase of neutrophils concentration obviously are the result of a stepwise inhibition of the differentiation of every type of neutrophils (by a feedback mechanism) and consequently leads to their accumulation during differentiation of B and MM and, therefore, to inhibition (regulation disturbance) of neutrophils maturation. In other words, S accumulation inhibits the maturation of B, and it in turn inhibits MM maturation. Finally, all neutrophils maturation orders are inhibited, increasing their concentration. At the same time, neutrophils inhibit Ph^+^ cells proliferation and PAmp;D efficiency. The next step, after reaching the "critical" neutrophils concentration, is characterized by apoptosis induction. It releases neutrophils from feedback, restores the initial neutrophils accumulation order, after which their concentration decreases, leading to restoration of the regulation of neutrophils accumulation, leading to increase of the P/D efficiency index and transition to effective proliferation of Ph^+^ cells.

The role of proliferation and maturation alternation. The alternation of effective proliferation and maturation with proliferation inhibitions that have different efficiencies and provide rate advantage to either the proliferation or maturation of neutrophils adds periodicity to this process. Efficient proliferation and myelocytes accumulation are synchronous to apoptosis induction, elevation of P/D^1^>1, but are asynchronous to neutrophils accumulation, inhibition of proliferation by them, inversion of the neutrophils accumulation order and changes of P/D^2^ within ≤ 1 (Fig. 2-4, Tables 1-3).

It should be noted that in the regulation of Ph^+^ cells PAmp;D, both synchronous and asynchronous processes could be observed. The apoptosis inhibition with neutrophils accumulation without dividing proceeds asynchronous to myelocytes accumulation and apoptosis induction. Myelocytes accumulation is synchronous to apoptosis induction, proliferation activation with increase of P/D index, and to restoration of maturation and proliferation regulation.

At the crossing points, PAmp;D parameters equalize. During types 1 and 2 Ph^+^ cells PAmp;D, its regulation at a given differentiation cycle becomes "irreversible." Modification of type 3 Ph^+^ cells properties is reversible: during stages alternation the properties inhibited at the previous stage can be restored. By PAmp;D regulation the type 3 Ph^+^ cells seems to be more "normal" than type 1 or 2. All types of Ph^+^ cells probably depend upon the inherited properties of the bcr/abl gene in different CML patients. Of note, the PAmp;D regulation of Ph^+^ cells in all three types of Ph^+^ cells from different CML patients differs in the quantitative characteristics of P/D efficiency, apoptosis inhibition, S accumulation, and the extent of Ph^+^ cells proliferation inhibition. Under PAmp;D of Ph^+^ cells types 1 and 2, its P/D efficiency indice varies in the limits P/D^1^>1 or P/D^2^ ≤ 1, correspondingly (Fig. 1 and 2, Table 1). It points to some extent of PAmp;D regulation of Ph^+^ cells in limits P/D^1^>1 or P/D^2^ without changing the initial advantage of the proliferation or maturation rate.

The differences in PAmp;D regulation are related to the properties of Ph^+^ cells from different CML patients at different phases of the disease. Evidently, PAmp;D efficiency is determined by myeloid cell precursors in Ph^+^ mononuclear cells fraction, inheriting the mutations in bcr/abl gene and p210 tyrosine kinase from different CML patients. The mutations capable of determining carcinogenicity in Ph^+^ cell lines, cell viability, activity of proliferation, and apoptosis blockage are studied in [7, 17, 18, 25, 27, 29, 30].

The alternation of Ph^+^ cells proliferation and maturation ex vivo with the rise or fall of PAmp;D efficiency participates in the regulation and maintenance of an optimum regimen of Ph^+^ cells PAmp;D. Under apoptosis blocking, it alternately switches inhibition of proliferation by maturing neutrophils, especially S, being the progeny of the same proliferating Ph^+^ cells. The cell regulation by segmented neutrophils probably mediates genetic regulation with participation of the bcr/abl gene. These results are concordant with evidence for the separate transforming and antiapoptotic activity of different mutant forms of p210 ^bcr/abl^ tyrosine kinase in cell lines that change signal transduction pathways [8, 9, 15,17, 18, 20, 27, 30, 31].

As a result, alternation of effective proliferation and effective maturation with the change of increased efficiency indices to very low ones leads to a fall in the overall PAmp;D efficiency of Ph^+^ cells types 2 and 3 and, thus, maintains a moderate, possibly optimum PAmp;D regimen. At maturation stages, PAmp;D regulation is mediated by the increase of neutrophils (especially S) content, proliferation inhibition with a decrease of P/D efficiency indices, and inversion of the neutrophils maturation order in Ph^+^ cells types 2 and 3.

## DISCUSSION

The elicited PAmp;D regulation in Ph^+^ cells ex vivo reveals the cellular aspects of genetic PAmp;D regulation. As was shown in the present work, cellular regulation is mediated by alternation of Ph^+^ cells proliferation with efficient maturation and is accomplished by proliferation inhibition by maturating without dividing neutrophils - progenies of the same proliferating Ph^+^ progenitor cells under conditions of apoptosis blockage.

The cellular regulation of hematopoietic cells PAmp;D and its proposed mechanism had previously never been considered in the literature [4-7, 9-12, 15, 16, 21- 23, 27, 30]. There was no data on the cellular regulation of Ph^+^ cells PAmp;D by mature neutrophils and on the alternation of stages with high and low PAmp;D efficiency until this study. Also, there was no data on the relative proliferation and maturation rate, their efficiency, and its quantitative equivalent (P/D index). The ratio of proliferating hematopoietic cells and mature neutrophils in single PB samples was designated previously as the maturation index [2].

The proliferation of myeloid progenitor cells and the initial stage of Ph^+^ cells PAmp;D regulation had been extensively studied [4,5,7, 8,10-12, 15,16, 20, 23-25, 26]. It is determined by the proliferative potential and concentration of CD34^+^ myeloid progenitor cells. Activation of Ph^+^ cells proliferation depends upon bcr/abl oncogene expression, its mutations, and higher rate of Ph^+^ stem cells transition from G_0_ to G_1_ cell cycle phase in comparison with cells without Ph chromosome [20, 23, 24]. Regulation of Ph^+^ cells proliferation is characterized to some extent (by the kinetic method of PAmp;D investigation) also in our study. It is clear from the PAmp;D parameters of type 1 Ph^+^ cells and alternations with effective proliferation stages in Ph^+^ cells type 3. The studies [33, 39] have shown the participation of CD34^+^ myeloid progenitor cells and bcr/abl expression in Ph^+^ cells from PB sample № 1.1, studied here in PAmp;D regulation of type 1 Ph^+^ cells. According to this, a maximum level of CD 34 antigen and bcr/abl expression is coincident with a high, efficient P/D index at the beginning of Ph^+^ cells PAmp;D.

The study [28] on Ph^+^ cells from CML CP patients isolated by flow cytometry has shown a direct relationship between myeloid Ph^+^ progenitors and bcr/abl oncogene expression. The authors have established a decrease in the proliferating Ph^+^ cells fraction in S+G2/M phases - by 4 times for myelocytes and by 7 times for B and S in comparison with CD34^+^ cells. A direct linear correlation of the Ph^+^ cells fraction in S+G2/M phases and bcr/abl expression was seen in CD34^+^ cells, myeloblasts, and promyelocytes. But later in the course of differentiation, the correlation inverted. It means, that active accumulation of proliferating Ph^+^ cells in S + G2/M phases at the beginning of the proliferation stage is proportional to the rise in bcr/abl expression, while during PAmp;D from myelocytes to B and S this proportionality inverses. In this study we didn't estimate kinetic parameters. The data used was obtained from a single probe of each of the 15 CML CP patients. Our results provide a good explanation of this correlation inversion in study [28] by a change in the mechanism of Ph^+^ cells proliferation due to inhibition of Ph^+^ cells proliferation by the neutrophils accumulating under apoptosis blockage.

Taken together, the work of Primo et al [28] and our study indicate the paradoxical mechanism of bcr/abl action on Ph^+^ cells. The expression of bcr/abl in proliferating myeloid Ph^+^ progenitors activates proliferation, but later mature Ph^+^ neutrophils that inherit the same oncogene bcr/abl inhibit proliferation. In other words, the promoting proliferation effect of bcr/abl oncogene expression in Ph^+^ cells at early stages of PAmp;D is inhibited by the expression of the same oncogene in the progenies of the same Ph^+^ cells at later stages of maturation. It can be explained, for example, by the change in the signal transduction pathways by bcr/abl oncogene, p210 tyrosine kinase, and spectrum of STAT proteins formed in the neutrophils or in one of them.

Some studies propose an alternative hypothesis on the development and progression of CML, considering the imbalance of stem cells self-renewal and further cell differentiation, rather than the activation of proliferation due to uncontrolled expression of p210 ^bcr/abl^, as the main cause of CML [20-24,13-14, 26]. It is associated with a decreased number of CML progenitor cells, while their progenies in CML are more abundant in comparison with that in "normal" cells. Distortion of Ph^+^ cells maturation as a primary biological defect in CML was discussed elsewhere rather long ago [13,14,21, 22, 42]. The example of Ph^+^ cells types 2 and 3 PAmp;D in our work shows that such an imbalance is mediated by the neutrophils inhibiting Ph^+^ cells proliferation at maturation stage under apoptosis blockage. The revealed differences in PAmp;D regulation with inhibition of proliferation by neutrophils with apoptosis blockage and alternation of efficient proliferation and efficient maturation of Ph^+^ cells from different patients inheriting different bcr/abl mutations completely explain the contradictions regarding the imbalance of Ph^+^ cells self-renewal and maturation.

Proliferation inhibition by neutrophils during the alternation of proliferation and maturation under apoptosis blockage is assumed to regulate also PAmp;D of hematopoietic cells in normal conditions. In CML, this mechanism can possibly be an attempt by CML cells to escape proliferation activation by bcr/abl expression, leading to CML progression. The threat of CML progression can be seen, for example, at PAmp;D of type 1 Ph^+^ cells under apoptosis induction and absence of neutrophils excess.

## CONCLUSIONS

1. The PAmp;D regulation of Ph^+^ cells from different CML patients is performed by alternation of effective proliferation with effective maturation. In the maturation stage, the Ph^+^ cells proliferation is inhibited by accumulating neutrophils with apoptosis blockage interrupting proliferation and maintaining the optimum PAmp;D level.

2. The alternation of stages consists in the switching of stage 1 - effective proliferation - when the proliferation rate of Ph^+^ cells exceeds that of maturation, to stage 2 - effective maturation - when apoptosis is blocked, and the neutrophils maturation rate is higher than the proliferation rate. The alternation can proceed according to the schemes 1/2 -1/2/1 or 2/1-2/1/2. The alternation stages have control points where its plots cross and the indices of proliferation and maturation are equal. The indices of P/D efficiency (ratio of proliferation and maturation rates) at control points are equal to 1.06±0.23 and don't depend upon time and alternation order, as well as the source of Ph^+^ cells - CML patients. In alternation stages, these indices are permanently changing.

3. In the proliferation stages, the proliferating cells content rises, the number of neutrophils (especially S) decreases and apoptosis is activated. In the maturation stages, on the contrary, apoptosis is blocked, the neutrophils content (especially S) is enhanced, while the number of immature is decreased. In the effective maturation stage, accumulating neutrophils inhibit the proliferation of immature Ph^+^ cells and the order (sequence) of neutrophils maturation is inversed and impaired, probably via a feedback mechanism.

4. The differences in PAmp;D regulation allowed us to identify 3 types of Ph^+^ cells in CML patients differing by the number and duration of alternation stages. There are Ph^+^ cells whose PAmp;D has either one prolonged effective proliferation stage or one prolonged effective maturation stage, or multiple alternations of effective stages of proliferation and maturation. The first corresponds to advanced CML phases; the other takes place in the CML chronic phase with good response to chemotherapy.

## Acknowledgements

This work was supported by the Russian Foundation for Basic Research (grant no. 06 04-08372-ofi).

## References

[1] Abdulkadyrov K.M., Bessmeltsev C.C., Rukavitsin O.A. (1998). Chronic myelogenous leukaemia..

[2] Vorobiev A.I., Newdiamed M. (2002). Haematological Guideline..

[3] Shiffman Blood Patophisiology.

[4] Deininger M.W.N., Goldman J.M., Melo J.V. (2000). Blood.

[5] Deininger M.W.N., Vieira S., Mendiola R. (2000). Cancer research.

[6] Medvedeva N.V. (2009). Haematological society. Clinical Oncohaematology.

[7] Melo J.V. (1996). Blood.

[8] Holyoake T.L., Jiang X., Eaves A.C., Eaves C.J. (2002). Leukemia.

[9] Holyoake T.L., Jiang X., Jorgensen H.G. (2001). Blood.

[10] Jamieson C.H.M., Ailles L.E., Dylla S.J. (2004). New England J Medicine.

[11] Jaiswal S., Traver D., Miyamoto T. (2003). Proc. Nat. Acad. Sci. USA.

[12] Passegue E., Jamieson C.H.M., Ailles L.E., Weissman I.L. (2003). Proc. Nat. Acad. Sci. USA.

[13] Strife A., Lambek C., Wisniewski D. (1983). Blood.

[14] Strife A., Lambek C., Wisniewski D. (1988). Cancer Res..

[15] Era T., Witte O.N. (2000). Proc. Nat. Acad. Sci. USA.

[16] Guzman M.L., Jordan C.T. (2004). Cancer Control.

[17] Bedi A., Zehnbauer B.A., Barber J. (1994). Blood.

[18] Bedi A., Barber J.P., Bedi G.C. (1995). Blood.

[19] Brandford S., Rudzki Z., Walsh S. (2002). Blood.

[20] Buckle A.M., Mottram R., Pierce A. (2000). Mol. Med..

[21] Clarkson B., Strife A., Perez A. (1993). Leukemia Amp; Limphoma..

[22] Clarkson B., Strife A. (1993). Leukemia..

[23] Coppo P., M L., Dusanter-Fourt I. (2003). Bonnet.

[24] Traycoff C.V., Haistead B., Rice S. (1998). Brit. J. Haematology..

[25] Lotem J., Sachs L. (1996). Leukemia..

[26] Lugo T.G., Pendergast A.M., Muller A.J., Witte O.N. (1990). Science..

[27] Cortez D., Kadlec L., Pendergast A.M. (1995). Mol. Cell. Biology..

[28] Primo D., Quijano S., Flores J. (2006). Brit.J. Haematology..

[29] Amarante-Mendes G.P., Naekyung K.C., Liu L. (1998). Blood.

[30] Selleri C., Maciejewski J.P., Pane F. (1998). Blood.

[31] Sherbenou D.W., Hantschel O., Turaga L. (2008). Leukemia..

[32] Stoklosa T., Poplawski T., Koptyra M. (2008). Cancer Res..

[33] Akhynina T.V., Gerasimova L.P., Sarkisyan G.P. (2007). Cytology..

[34] Abramov M.G. (1985). Haematological album. M: Medicine.

[35] Gerasimova L.P., Manakova T.E., Akhynina T.V. (2002). Russian Journal of Biotherapy..

[36] Pinegin B.V., Yiarilin A.A., Simonova A.V. (2001). Apoptosis evaluation of human peripherial blood activated lymphocytes by cytofluorimetric method with propidium jodide. Use of flow cytofluorimetry for estimation of human immune system functional activity.. MH. RF.

[37] Shmarov D.A., Kosinets G.I. (2004). Methods of cell cycle analysis by flow cytofluorimetry. Medical Informational Agency.

[38] Dean P.N. (1980). Cell Tissue Kinet..

[39] Grineva N.I., Barishnicov A.Ju., Gerasimova L.P. (2007). Russian Journal of Biotherapy..

[40] Kosinets G.I., Kotelnicov V.M. (1983). Soviet Medicine..

[41] Kotelnicov V.M., Kosinets G.I., Kasatkina V.V., Kovalevskaya N.P. (1982). Kinetics of granulocytepoiesis.. Kinetic aspects of haemopoiesis.

[42] Golde D.W., Cline M.J. (1973). New Engl. J. Med..

[43] Vladimirskaya E.B. (2001). Mechanisms of blood cells apoptosis.. Laboratorial Medicine..

[44] Vladimirskaya E.B. (2001). Apoptosis and its role in the development of tumor growth. Biological basis of antitumour therapeutics.. Agat-Med.

[45] Dublez L., Eymin B., Sordet O. (1998). Blood.

[46] Goldman J.M., Th'ng K.G., Catovsky D., Galton D.A.D. (1976). Blood.

